# Virtual Surgical Planning and Patient-Specific Instruments for Correcting Lower Limb Deformities in Pediatric Patients: Preliminary Results from the In-Office 3D Printing Point of Care

**DOI:** 10.3390/jpm13121664

**Published:** 2023-11-28

**Authors:** Giovanni Trisolino, Alessandro Depaoli, Grazia Chiara Menozzi, Luca Lerma, Michele Di Gennaro, Carmelo Quinto, Leonardo Vivarelli, Dante Dallari, Gino Rocca

**Affiliations:** 1 Unit of Pediatric Orthopedics and Traumatology, IRCCS Istituto Ortopedico Rizzoli, 40136 Bologna, Italy; giovanni.trisolino@ior.it (G.T.); alessandro.depaoli@ior.it (A.D.); luca.lerma@ior.it (L.L.); michele.digennaro@studio.unibo.it (M.D.G.); gino.rocca@ior.it (G.R.); 2 Reconstructive Orthopaedic Surgery and Innovative Techniques—Musculoskeletal Tissue Bank, IRCCS Istituto Ortopedico Rizzoli, 40136 Bologna, Italy; carmelo.quinto@ior.it (C.Q.); leonardo.vivarelli@ior.it (L.V.); dante.dallari@ior.it (D.D.)

**Keywords:** pediatric orthopedic surgery, virtual surgical planning, 3D printing, limb deformity

## Abstract

(1) Background: Virtual reality and 3D printing are transforming orthopedic surgery by enabling personalized three-dimensional (3D) models for surgical planning and Patient-Specific Instruments (PSIs). Hospitals are establishing in-house 3D printing centers to reduce costs and improve patient care. Pediatric orthopedic surgery also benefits from these technologies, enhancing the precision and personalization of treatments. This study presents preliminary results of an In-Office 3D Printing Point of Care (PoC), outlining considerations and challenges in using this program for treating lower limb deformities in pediatric patients through Virtual Surgical Planning (VSP) and 3D-printed Patient-Specific Instruments (PSIs). (2) Materials and Methods: Pediatric patients with congenital or acquired lower limb deformities undergoing surgical correction based on VSP, incorporating 3D-printed PSIs when required, were included in this study. The entire process of VSP and 3D printing at the In-Office PoC was illustrated. Data about deformity characteristics, surgical procedures, and outcomes, including the accuracy of angular correction, surgical times, and complications, were reported. (3) Results: In total, 39 bone correction procedures in 29 patients with a mean age of 11.6 ± 4.7 years (range 3.1–18.5 years) were performed according to VSP. Among them, 23 procedures were accomplished with PSIs. Surgeries with PSIs were 45 min shorter, with fewer fluoroscopy shots. Optimal correction was achieved in 37% of procedures, while the remaining cases showed under-corrections (41%) or over-corrections (22%). Major complications were observed in four patients (13.8%). (4) Conclusions: The In-Office 3D Printing Point of Care is becoming an essential tool for planning and executing complex corrections of lower limb deformities, but additional research is needed for optimizing the prediction and accuracy of the achieved corrections.

## 1. Introduction

Virtual reality and 3D printing are rapidly advancing in various surgical fields, especially orthopedic surgery. The ability to create three-dimensional (3D) bio-models of a patient’s anatomy and use them for surgical planning and simulation, as well as producing personalized implants and instruments, has become essential in modern surgery. Many hospitals have responded by establishing in-house surgical simulation and 3D printing centers to promote and expand the use of these technologies, reducing turnaround times and costs. This aligns with the growing demand for personalized medicine and tailored surgical solutions. New production models like “point-of-care (POC) manufacturing” allow hospitals to meet spatial requirements, consolidate technical expertise, and keep their service offerings up to date without relying on quickly outdated machinery and materials, which is common in this field. Pediatric orthopedic surgery is not immune to these technological advancements. Pediatric orthopedic surgery addresses congenital and acquired limb and spine deformities in children, focusing on restoring function, enhancing aesthetics, and ensuring proper limb and spine growth. It employs techniques like osteotomies, bone grafts, and age-specific or custom-made hardware. While some deformities are straightforward, pediatric orthopedics often encounter complex rare deformities and non-standard clinical cases. Treating such complexities demands extensive preoperative planning due to the intricate 3D anatomy of the axial and appendicular skeleton.

Virtual Surgical Planning (VSP) is part of Computer-Assisted Surgery (CAS), which emerged in the late 1980s and aimed to develop new approaches to support surgeons in performing surgeries by exploiting the computational power of computers [1,2,3,4]. VSP is the process of simulating and planning a surgical correction entirely within a virtual environment, regardless of whether it is immersive (fully immersive virtual reality) or non-immersive (computer-based simulations). VSP supports surgeons in the definition of various aspects of the surgery, including three-dimensional analysis of the deformity, selection and placement of implants, determination of access points, increasing the customization in the preoperative planning of the intervention, intraoperative execution, and postoperative evaluation.

The analysis of a unifocal bone deformity must evaluate all its different possible components: (1) angulation, defined as the inclination between the proximal and distal axes of a bone segment on a plane of maximum deformity between the coronal and sagittal planes; (2) torsional deformity, defined as the angle of rotation in the longitudinal axis between the proximal and distal segments; and (3) translation, which can be divided into the components of shortening/elongation and possible ad latus translation [5]. While angulation can be easily estimated on simple radiographs, evaluation of translations requires accurate radiographic studies with landmarks, and imaging evaluation of rotations requires the use of CT or MRI techniques to assess torsional axes.

The execution of virtual plans can achieve greater refinement and precision by producing and utilizing patient-specific tools created through 3D printing [6,7,8,9,10]. Personalized 3D-printed tools, including patient-specific models, guides, instruments, and implants, offer several advantages: they reduce surgery time, minimize blood loss, shorten incision length, and require less bone resection. The complete sequence of surgical simulation, VSP, design, and 3D printing of PSIs can be executed within designated “in-hospital” 3D-printing point-of-care (POC) facilities. Hospitals have increasingly established in-office 3D-printing facilities to improve efficiency and cut costs, but successful implementation involves multiple steps, from imaging to physical model creation, requiring careful oversight.

In this report, we present a single-institutional case series involving pediatric patients with complex lower limb deformities undergoing VSP in all cases presented and PSI-assisted surgery in a part of them. This article aims to share our hospital-based 3D printing experience at POC, outlining considerations and challenges in initiating or expanding such a program.

## 2. Materials and Methods

### 2.1. Study Design and Patient Selection

This article analyzes the preliminary results of a prospective clinical trial which is still recruiting pediatric patients with congenital or acquired limb deformities who received treatment based on VSP, incorporating 3D-printed PSIs when possible. Patients were considered eligible if they satisfied the following criteria: (1) underwent Virtual Surgical Planning (VSP) for one or more segment of the long bones of the lower limbs; (2) had accessible data regarding planned correction parameters; (3) underwent the planned surgical correction; and (4) provided informed consent for data processing. Exclusion criteria encompassed: (1) planned corrections not executed; (2) missing data pertaining to the planned surgical correction.

### 2.2. VSP and 3D-Printing Procedure at the In-Hospital 3D-Printing POC

**Figure 1:**
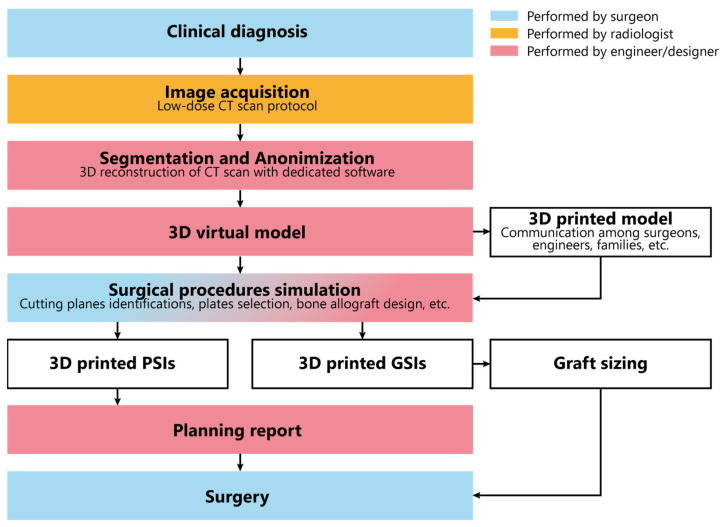
In-Office 3D Printing PoC workflow.

The In-Office 3D-Printing PoC Workflow is illustrated in Figure 1.

**Figure 2:**
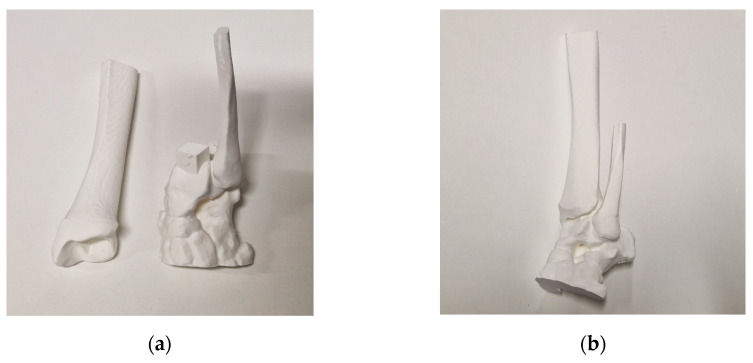
(**a**) Disassembled 3D model of an ankle with the connecting pin placed on the talus; (**b**) 3D model assembled.

The process starts with image acquisition using low-dose CT scans for assessing limb deformities. When possible, we incorporate the healthy contralateral limb to facilitate mirroring and deformity correction. In cases involving multiple segments, all relevant segments are included in the CT scans, such as the thigh, leg, and foot. The obtained images undergo anonymization and segmentation through dedicated software (Mimics, Materalise 25.0). Three-dimensional digital models are obtained by image reconstruction and conversion into Standard Triangulation Language (STL) format. The digital models are 3D printed to enhance tactile understanding and improve communication between surgeons and engineers. They are also employed to explain deformities and procedures to residents, students, and families. In cases of the entire long bones, the 3D printing process must consider the maximum build size of the printer. Therefore, the print needs to be divided into multiple segments. Attachment pegs and holes are manually added to the segmented 3D file for easy reassembly after printing (Figure 2).

**Figure 3:**
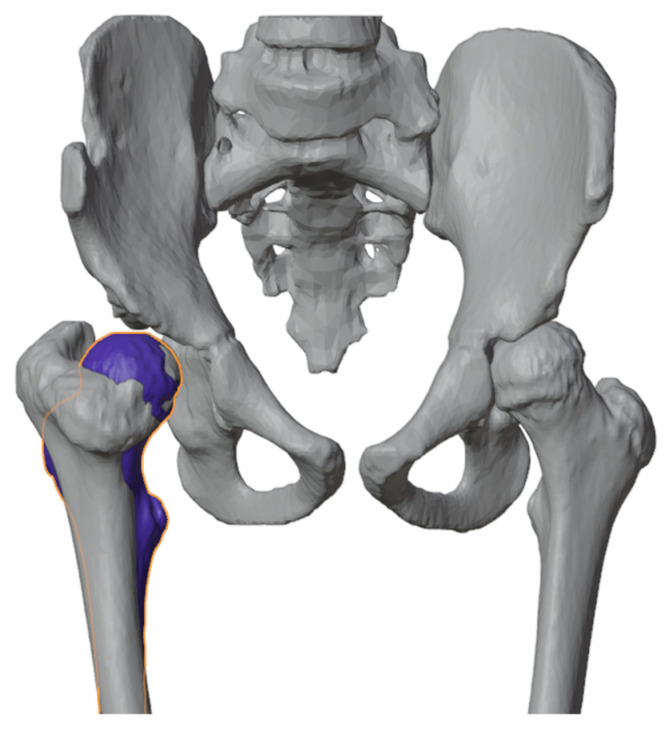
Healthy proximal femur (blue femur) mirrored and superimposed on the one to be corrected.

**Figure 4:**
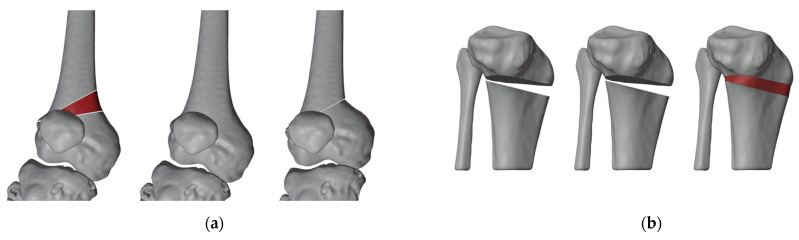
(**a**) Planning of bone graft (red part of the model) harvesting for closing-wedge osteotomy; (**b**) bone graft shaping (red part of the model) for opening-wedge osteotomy.

**Figure 5:**
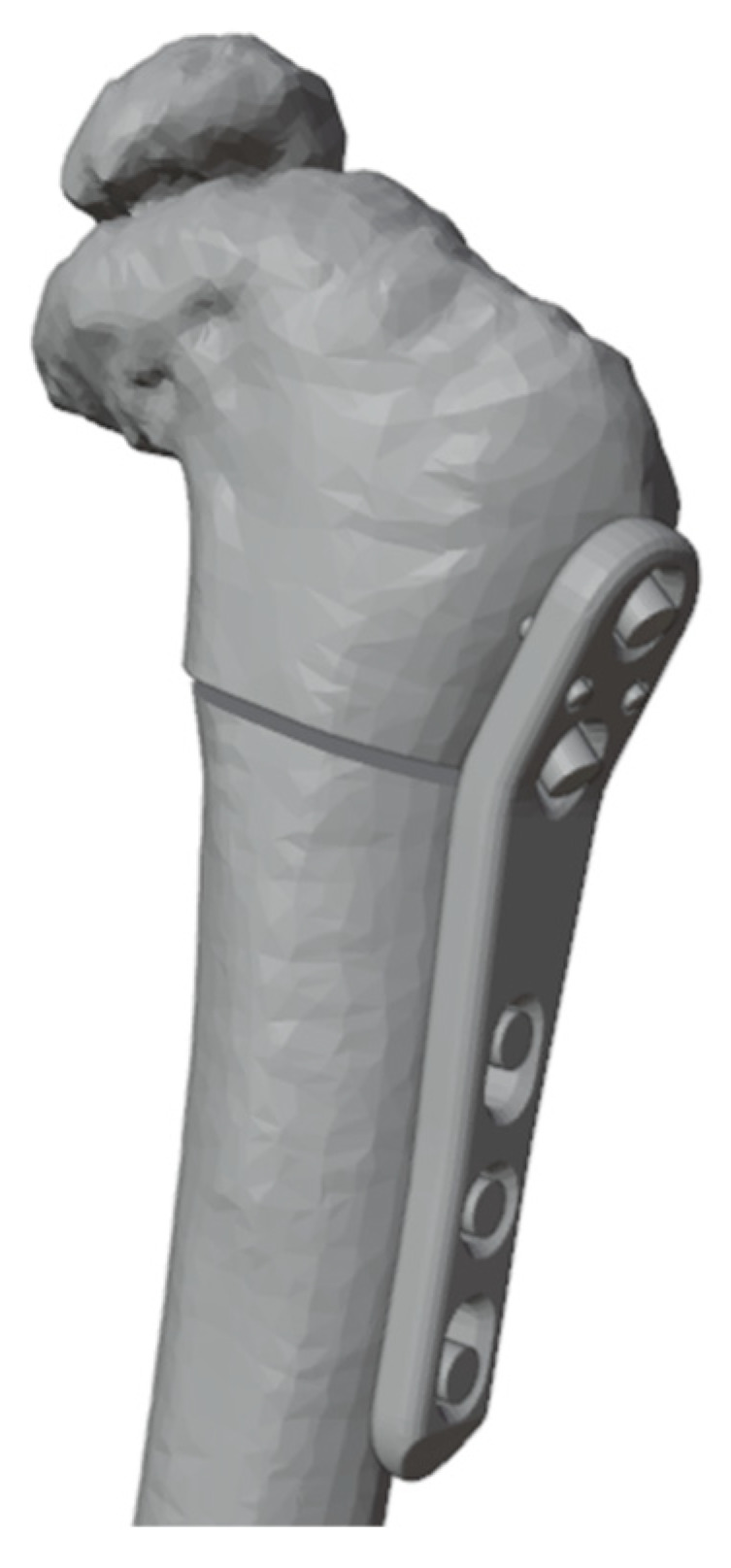
Selected fixation device placed with placeholders to identify screws.

On the digital model, the surgical procedure is simulated in a non-immersive virtual environment. This simulation process is accomplished throughout various techniques: (1) overlaying the image of the healthy side (where possible) to achieve a correction that mirrors the unaffected limb (Figure 3); (2) applying precise angular correction values, derived from the analysis of the segment’s deformity and the normal values for that specific segment (if both limbs are affected); (3) evaluating the position, type, and orientation of osteotomies as required; (4) examining the dimensions of removed bone segments (for closing-wedge osteotomies) or the dimensions and shape of bone grafts (for opening-wedge osteotomies) (Figure 4); (5) selecting the most suitable fixation device, ensuring accurate sizing and proper placement of the chosen hardware (Figure 5). The entire simulation process is conceived, monitored, and approved by both the surgeon and the engineer.

**Figure 6:**
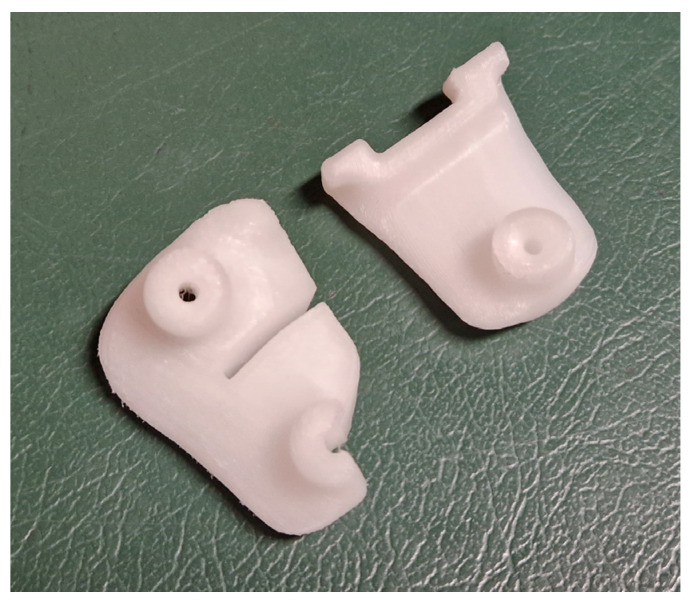
Example of guide design for the use of instrumentation employed with different directions (**left**) and for the use of instruments being used along the same direction (**right**).

**Figure 7:**
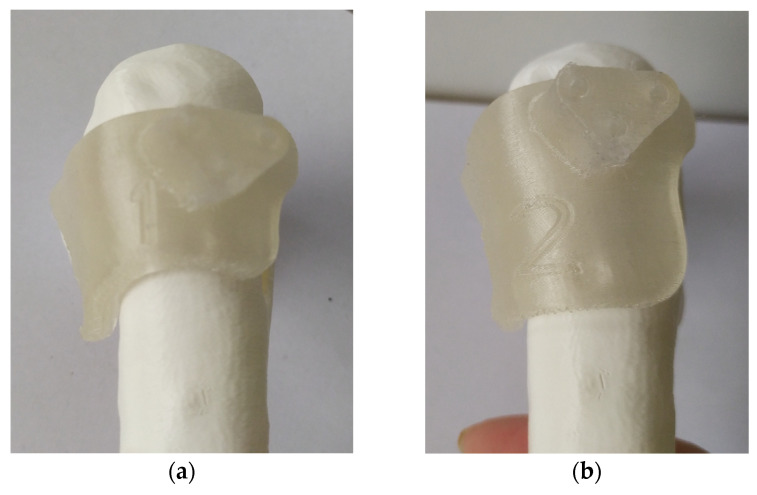
Embedded numbers in the guides: (**a**) first mask to be used during the procedure; (**b**) second mask to be used for the procedure.

Upon approval of the surgical plan, when necessary, PSIs including cutting jigs, templates, and guides are designed. It is imperative to consider the preferred surgical approach and any potential constraint posed by inviolable anatomical structures. The template design should consider size, shape, and position of any hardware (e.g., plates, screws), instruments (e.g., saws dimensions, wires), and osteotomy levels, directions, and orientations. Guides should be secured with suitable K-wires (usually 2 mm), accommodating rotational correction when needed and enabling reverse correction application (starting from the desired final correction and then working backward to the initial deformity). If divergent wires are expected, design the guides with semi-open tunnels for easy removal (Figure 6). When using multiple similar guides during the same procedure, embossing numbers and letters is recommended to facilitate use and prevent positioning errors during surgery (Figure 7).

**Figure 8:**
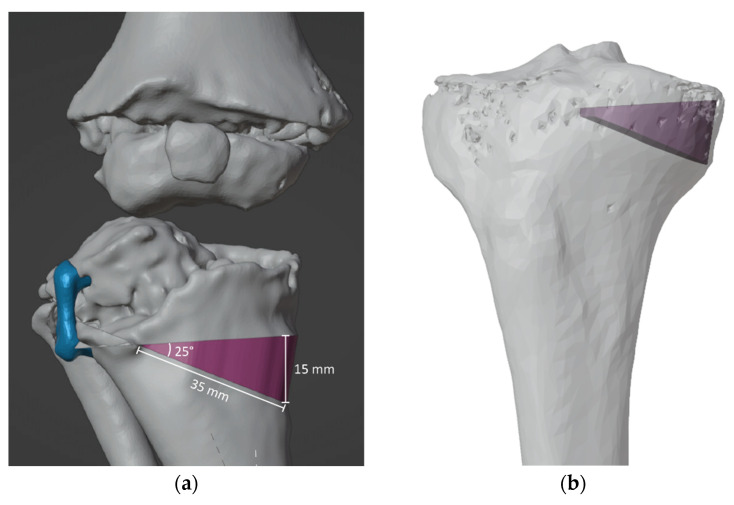
(**a**) Custom bone allograft identification (purple) with VSP on previous emiepiphyseal stapling with 8-plate (blue), (**b**) Custom bone allograft identified (purple) is used to perform donor bone matching.

The templates are then 3D printed using HT-PLA on an FDM 3D printer, following a previously described procedure, and sent to the hospital pharmacy service for steam sterilization. A comprehensive report is also generated, encompassing the entire procedure, including the various steps for utilizing the guides, the precise measurements for osteotomies, and the hardware required (Supplementary Materials Document S1). This is aimed at streamlining their utilization, even for the operating room staff during surgery. When necessary, customallografts are prepared by matching the desired graft shape with the most suitable donor bone, allowing us to harness the biomechanical properties of structural allografts (Figure 8). The preparation methods for custom bone grafts have been detailed in a prior study [11,12].

### 2.3. Collected Data

After obtaining informed consent, demographic information (age, gender, height, weight, adjusted BMI, underlying pathology, and comorbidities) were recorded. Preoperative X-rays and CT scans were used to document relevant radiographic details, image quality, and the time interval between CT imaging and surgery. CT images were assessed as suboptimal in case of presence of artifacts, low image resolution, absence of the contralateral skeletal segment, and/or partial acquisition of the skeletal segment of interest.

Surgical variables encompassed procedure type, number, and location of skeletal segments treated, correction method, hardware utilization, PSI usage, bone graft type (if employed), PSIs, customized bone graft, operation duration, fluoroscopy, intraoperative bleeding, and perioperative complications classified by the Clavien Dindo Sink classification modified by Dodwell et al. (mCDS) [13]. Complications up to grade 2 were categorized as minor, while complications graded 3 to 5 were classified as major. The set of procedures performed through a single surgical incision was counted as one segment correction. When performing surgery on multiple segments simultaneously, we divided both the surgical time and the number of fluoroscopy shots by the number of segment corrections. Segment corrections were divided into two groups: procedures with only VSP and procedures with VSP and PSIs. Moreover, the planned correction of angular deformity on VSP was assessed and compared with the actual angular correction achieved during surgery, both measured on the true plane of deformity. The planned correction (PC) angles were readily available in the surgical planning reports provided to orthopedic surgeons for procedure summarization and, when necessary, to illustrate the application of PSIs. The angles of achieved correction (AC) were estimated by two authors (A.D. and M.D.G.) based on postoperative radiographs taken within one to five days after the surgery. In cases where radiographs on the true plane of angulation were unavailable, the actual angulation was calculated using the Ilizarov triangle method [14]. Subsequently, the PC angle was subtracted from the AC angle to determine the difference from the intended correction. A value of −3° or less was considered an under-correction, while a value of +3° or more was regarded as an over-correction. An angle within the range of −2° to +2° was considered an optimal correction. To assess the overall accuracy of the angular correction of the procedures, the error of correction was quantified as the absolute difference between AC and PC, expressed in degrees (°) and as a percentage (%).

$$Angular\;correction\;difference\left({}^{\circ}\right)=AC-PC$$

$$Angular\;correction\;error\left({}^{\circ}\right)=|AC-PC|$$

$$Angular\;correction\;error\left(\%\right)=\frac{\left|AC-PC\right|}{PC}\times 100$$

The following variables were considered outcome measures: angular correction error, surgical time per number of segment corrections, number of intraoperative fluoroscopy images, and the number of minor and major complications.

### 2.4. Statistical Analysis

Data were collected using Excel 2022 (Microsoft Corporation, Redmond WA, USA) and then transferred on STATA (StataCorp. 2022. Stata Statistical Software: Release 17. College Station, TX, USA: StataCorp LLC.) to perform statistical analysis.

Spearman’s test for nonparametric variables was performed, and possible correlations that emerged were evaluated with subsequent uni- or multivariate linear regressions for values of *p* < 0.1. For categorical variables, frequency, and distribution according to anatomical district and type of osteotomy were evaluated; for continuous variables, means, standard deviations, and ranges were evaluated. A comparison of discrete variables was assessed by constructing contingency tables, considering an adjusted residual > 2.0 as significant. The difference of continuous variables between groups was evaluated by comparing means. A *p* value < 0.05 was considered significant.

## 3. Results

### 3.1. Patients’ Demographics and Baseline Variables

Between January 2018 and May 2023, 67 patients underwent digital 3D model reconstruction of one or more skeletal segments. Of these, 23 received 3D model reconstruction solely for visualization, and were excluded, while 14 underwent procedures planned by VSP in segments other that long bones of lower limbs and were also excluded, one patient underwent VSP and PSI design, but the surgical procedure was not performed for traumatic fracture at the site of planned osteotomy. The remaining 29 patients underwent VSP for 32 surgical procedures, for a total of 39 bone segment corrections planned for deformity of the long bones of the lower limb. A total of 16 procedures were performed with only the VSP and 23 with VSP and PSIs. The mean age at surgery was 11.6 ± 4.7 years (range 3.1–18.5 years) and the mean follow-up was 0.7 ± 0.8 years (range 2 months–3.4 years). One patient underwent VSP and PSIs for the precise placement of a screw–plate to address an impending fracture in a unicameral bone cyst of the femoral neck, while the remaining 38 procedures were acute correction osteotomies. Details of the type of osteotomy by district are described in Table 1.

### 3.2. Preoperative CT Results

A total of 29 CT studies were performed in 29 patients to plan 39 bone segment corrections; 11 CT studies (38%) were performed according to low-dose Veo protocol; 24 CTs were executed at our institution, while 5 were performed elsewhere; 11 of 29 CTs (38%) were rated as “suboptimal” but were still used to plan 15 corrections. None of the patients who underwent more than one surgery needed an additional CT study between the first and second operations. With the numbers available, we found no significant correlations between the appropriateness of the radiographic study and the quality of the correction achieved. 

The mean number of days between the CT study and surgery is 162 ± 146 days (range 2–521). The interval between imaging and surgery showed no impact on the accuracy of the correction (*p* = 0.99).

### 3.3. Corrections Results Related to PSIs Use

**Figure 9:**
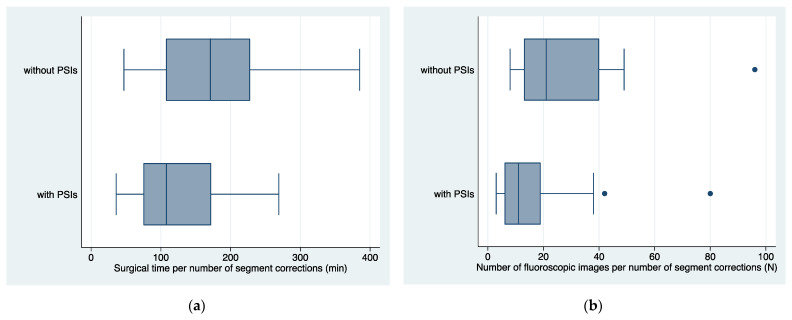
(**a**) Boxplots of surgical times per number of segment corrections in femur or tibia with and without PSIs; (**b**) boxplots of average number of fluoroscopic images per number of segment corrections in femur or tibia, with and without PSIs. PSIs = Patient-Specific Instruments produced in the In-Office 3D Printing Point of Care; min = minutes; N = number.

The average time for single correction was 145 ± 79 min (36–385). The number of fluoroscopic shots was 22 ± 21 (3–96). Surgeries aided by PSIs were notably shorter, averaging 125 ± 16 min (C.I. 93–157), compared to those without PSIs at 170 ± 19 min (C.I. 131–209). This resulted in a time savings of approximately 45 min (C.I. 95% from −95 to +5 min), representing about 26% of the total surgical time (*p* = 0.078) (Figure 9a). The average fluoroscopy shots needed without PSIs were 29 ± 5, compared to 16 ± 4 shots with PSIs, resulting in a mean difference of 13 ± 7 shots (95% CI from −26 to −1 shots, *p* = 0.049, Figure 9b).

**Figure 10:**
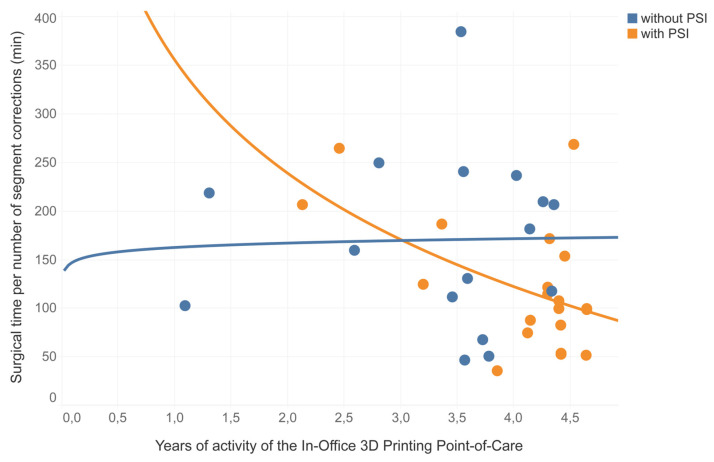
Representation of logarithmic correlations of the surgical time per number of segment corrections, distribution of femur and tibia corrections during the clinical trials years, divided between procedures performed without PSIs (in blue) and procedures performed with PSIs (in orange). PSIs = Patient-Specific Instruments produced in the In-Office 3D Printing Point of Care; min = minutes.

Through a time-based analysis of the data, it was observed that the duration of femoral and tibial osteotomies assisted by PSIs exhibited a decreasing trend following a logarithmic pattern (*p* = 0.01), whereas corrections without PSIs demonstrated consistent, stable values over time (Figure 10).

**Figure 11:**
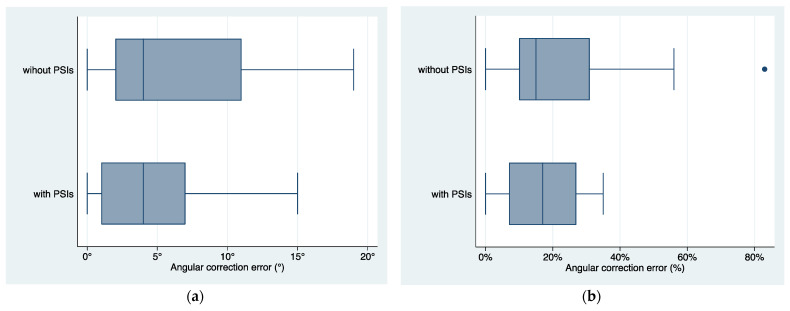
Boxplots of distribution of angular correction error (**a**) in degrees (°) and (**b**) in percentage (%) in femur or tibia corrections performed with and without PSIs. PSIs = Patient-Specific Instruments produced in the In-Office 3D Printing Point of Care.

The evaluation of angular correction accuracy was conducted on 27 segments, representing 69% of the entire series. Out of these, the average planned angular correction was 26° ± 3° (range 3°–74°). The mean achieved angular correction was 26° ± 13° (range 3°–60°). The mean angular correction error was 4.9°± 4.7° (20.7% ± 18.4%). The mean angular correction error was 6.0° ± 5.7° (25.4% ± 24.9%) in procedures performed without PSIs and 4.1° ± 3.8° (17.3% ± 11.3%) in procedures performed with PSIs with no statistically significant difference (*p* > 0.28, Figure 11).

In total, 10 corrections (37%) achieved the optimal angle, while 11 were under-corrected (41%), deviating more than −2° from the planned correction, and 6 were over-corrected (22%), exceeding more than 2° above the intended correction. 

### 3.4. Complications

A total of 14 patients (48%) had one or more complications. Of them, four patients (14%) experienced major complication. Two patients had a deep infection requiring surgical debridement and prolonged intravenous antibiotic therapy, two further patients treated for congenital pseudarthrosis of tibia had non-union at the site of osteotomy. A weak correlation was found between the time of surgery and the mCDS degree of the most serious complication (R^2^ = 0.24, *p* = 0.001) and with the major complications (R^2^ = 0.23 *p* = 0.01). The analysis estimated that after the first 111 ± 14 min of surgery, the mCDS of the most serious complication increases by 1 every 36 ± 10 additional minutes.

Among patients affected by congenital pathologies, 71% experienced one or more complications, whereas 27% of those with acquired pathologies encountered one or more complications (*p* = 0.03). 

## 4. Discussion

This work updates our preliminary experience with VSP for acute lower extremity deformity corrections, focusing on the influence of PSIs produced through the In-Office 3D Printing PoC with FDM technology using HTPLA. We extensively incorporated VSP and 3D-printed PSIs throughout the preoperative and operative phases. We confirmed that having an In-Office 3D Printing PoC greatly boosts the adoption of VSP and PSIs, providing remarkable versatility in pediatric orthopedic surgery. It also enhances surgeons’ confidence and usability, while also reducing costs compared to third-party vendors and companies. In the first 5 months of 2023, at our office, 25 surgical procedures were performed using 3D models, VSP, and PSIs, averaging approximately 1.2 procedures per week. This minimum threshold was estimated to cover annual fixed costs [15]. The presence of a highly skilled and specialized engineer in the team, along with high-performance dedicated computers, software licenses, and a suitable environment for positioning the 3D printer, are fundamental prerequisites for establishing an In-Office 3D Printing PoC.

Despite limitations like limited follow-up, the heterogeneity of regions and conditions, and the evaluation concentrated solely on angular correction, some interesting data emerged. 

Overall, our results confirm that on-site 3D printing of PSIs for VSP-assisted surgery is a safe and effective method for achieving precise corrections in pediatric orthopedic procedures, particularly in lower limb long bone corrections. This approach significantly reduces surgical duration and the need for intraoperative fluoroscopy, as reported in previous studies [16,17,18,19]. Zheng et al. found an average decrease in surgical time between exposure of the greater trochanter and definitive plate fixation from 47 min to 21 min, comparing proximal femur osteotomies performed with conventional technique and using cutting guides [17]. In our study, we showed a significant average time saving of 45 min per procedure using PSIs, mainly due to decreased reliance on intraoperative fluoroscopy. This becomes more noteworthy as surgical times decrease with growing confidence in PSI utilization.

Our study highlights that shorter surgical times in these complex procedures are crucial, not only for reducing complications and enhancing surgical performance but also for enabling simultaneous corrections across various body regions, thus reducing the need for multiple surgical events. The substantial reduction in intraoperative fluoroscopic images is a noteworthy and well-documented indirect indicator of complications during surgery. Numerous authors have reported similar findings, highlighting the decreased reliance on radiographic shots when using PSIs [17,18,19].

The literature underscores that prolonged surgical times increase the risk of complications, with a 20% increase estimated to elevate the odds of surgical site infections in orthopedic surgery by 3.6 to 7.4 times [20]. Our case series reveals a noteworthy correlation: an increase in surgical time corresponds to both a higher number and severity of complications. However, potential confounding factors should be considered. All procedures in the study are of medium to high complexity, inherently carrying a heightened complication risk. Consequently, the heterogeneity of procedures performed may contribute to a higher complication prevalence, especially in more complex cases requiring longer surgical times. It is important to emphasize that in our experience, we primarily employed VSP for extensive corrective osteotomies (averaging 26° on the maximum plane of deformity) mainly in congenital deformities, which inherently carry a heightened risk of complications, irrespective of the use of VSP and PSIs. Acute bony corrections in the lower extremities beyond a certain limit are frequently discouraged due to the potential risk of complications. Gradual corrections using external fixators are preferred but also come with their own set of complications and can significantly impact the child’s psychological well-being. So, the question is whether this significant number of complications is primarily a result of overconfidence in VSP, driving us to pursue more radical and original solutions, or are they predominantly linked to the patient’s underlying conditions and the inherent challenges of the underlying pathology. Our current study is not designed to definitively answer this question. Ongoing comparative studies on large cohorts of patients will determine whether complication rates are associated with the use of VSP and PSIs or the selection of more complex surgeries.

We observed a significant prevalence of suboptimal angular corrections, with a greater prevalence of under-corrections than over-corrections. This finding was unexpected, given surgeons’ typically high satisfaction with intraoperative results following VSP implementation. The literature reports angular correction accuracy using PSI within a range of 0.3° ± 2.1° to 1.0° ± 0.9° [21,22]. However, we question the reliability of these studies’ methodology, as they fail to consider absolute values, leading to offsetting under-corrections and over-corrections. Applying a similar analysis to our study data, the average error would be 0.6° ± 10°, aligning with the reported findings.

In our analysis of two-dimensional data, we observed that, despite using PSIs, VSP consistently yields an absolute deviation margin of approximately 4° to 6°, corresponding to 17–25% between planned and actual corrections. For instance, if we plan a 20° bone correction on a plane, the final angle may deviate by nearly 3°–5° from the planned correction. This margin of error, though significant, can be attributed to various factors, including not only the planning and implementation of PSIs but also technical challenges during the procedure and the natural progression of certain conditions in growing individuals. This aspect warrants further evaluation, particularly in terms of long-term functional outcomes, and we suggest integrating a satisfaction parameter into post-surgery surveys to gain deeper insights.

Despite the wide number of procedures included, several limitations must be considered. 

First, the high heterogeneity of conditions and age among the patients may be a potential confounding factor. For this reason, we decided to focus the analysis on the perioperative outcomes, since functional outcomes and/or PROMs results were not comparable among different conditions. Further research is needed to gather wide cohorts of comparable patients and homogeneous surgical treatments.

Second, although the adoption of VSP has gradually gained more favourable acceptance among our surgeons, with an increasing demand for its use in resolving more complex surgical cases, our current research cannot conclusively establish the precise advantages of VSP over the sole traditional two-dimensional methods of planning, especially in pediatric orthopedics. Our study lacks a control group of conventionally treated patients. Surgical plannings included in this study treat deformities that are often severe, rare, and in some cases, associated with skeletal dysplasia. A retrospective comparison with procedures performed only by conventional planning on plain radiographs would be interesting. However, it is scarcely possible to trace data on corrections planned in the traditional way. As a result, a comparison is not possible, whereas with 3D, we have all the planning reports. Additional well-designed case–control studies or prospective randomized clinical trials are essential to conclusively establish the efficacy of VSP and PSIs compared to traditional bidimensional planning methods.

Third, VSP is specifically designed to enable three-dimensional multi-focal or multi-segment corrections, enhancing precision and confidence, particularly in cases with narrow skeletal dimensions. Therefore, a comprehensive evaluation of correction outcomes should include postoperative CT scans, which are not commonly part of routine clinical practice, especially in pediatric patients.

## 5. Conclusions

This study supported the efficiency of Virtual Surgical Planning (VSP) in reducing surgical duration and minimizing fluoroscopy usage when coupled with Patient-Specific Instruments (PSIs). Although further research is essential for precise benefit quantification, including cost analysis, it becomes increasingly evident that those seeking to leverage these technologies should equip themselves with a PSI 3D printing facility, such as an In-Office 3D Printing Point of Care.

## Figures and Tables

**Table 1:** Number and district of acute corrections performed by type of osteotomy.

Skeletal District	Shortening Translation	Opening Wedge	Intercalary Graft	Dome + Opening Wedge	Total
Proximal femur Distal femur	18 5	- 1	- 1	- -	18 7
Proximal tibia Tibial shaft Distal tibia	- - 5	1 - -	- 2 -	5 - -	6 2 5
**Total**	**28**	**2**	**3**	**5**	**38**

## Data Availability

Data are available from the corresponding author upon reasonable request.

## Supplementary Materials

The following supporting information can be downloaded at: https://www.mdpi.com/article/10.3390/jpm13121664/s1, Document S1: Report–EXAMPLE.
