# Supplementary material for: Virtual Surgical Planning and Patient-Specific Instruments for Correcting Lower Limb Deformities in Pediatric Patients: Preliminary Results from the In-Office 3D Printing Point of Care
Source: J Pers Med. 2023 Nov 28;13(12):1664. doi: 10.3390/jpm13121664 (PMC10745053; doi:10.3390/jpm13121664)

PLANNING REPORT

Patient Name: Name: XXXXXXX Surname: XXXXXXX

Patient ID: IDXXXXXX

Surgery date: ☒☒ / ☒☒ / ☒☒☒☒

Surgeon: Dott. XXXXXX

Bone graft: ☒ Yes ☐ No

Donor ID IDXXXXXX

Hardware required: 3,5 mm cannulated screws

Other:

Surgery notes:

Planned PSI: 1

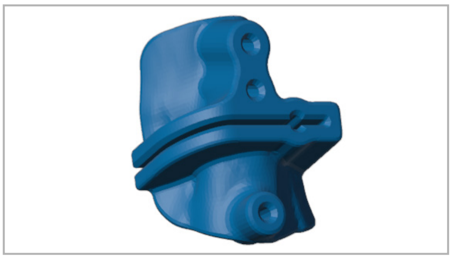

Mask ID:
IDXXXXXX_MTD

Planning references:

IDXXXXXX_MTD placement, proximal Ø2mm K-wire insertion, and ampliscopic control

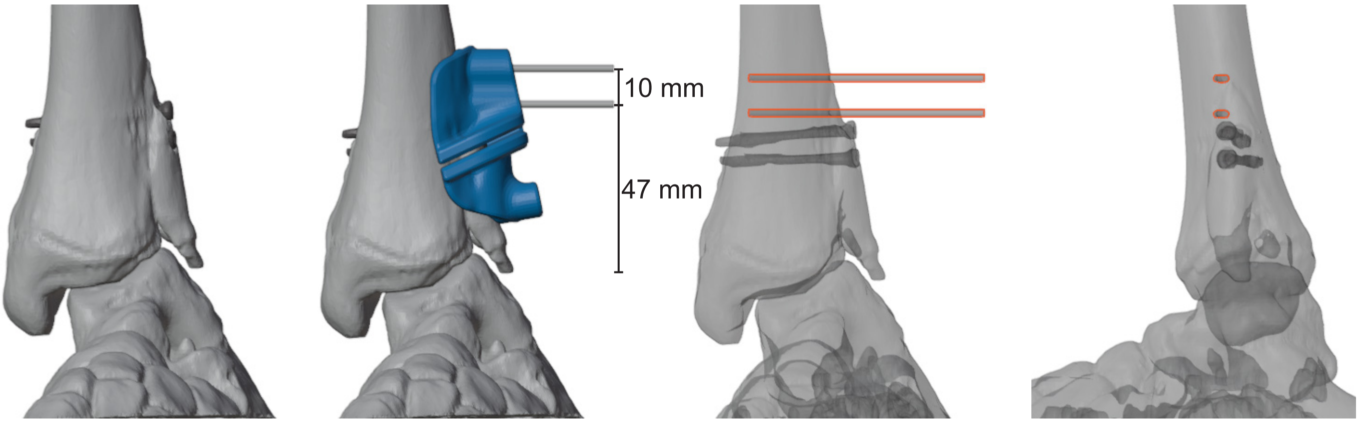

IDXXXXXX_MTD removal for hardware removal, IDXXXXXX_MTD reinsertion on proximal K-wires and distal K-wire insertion (if desired, also K-wire for cutting), ampliscopic control

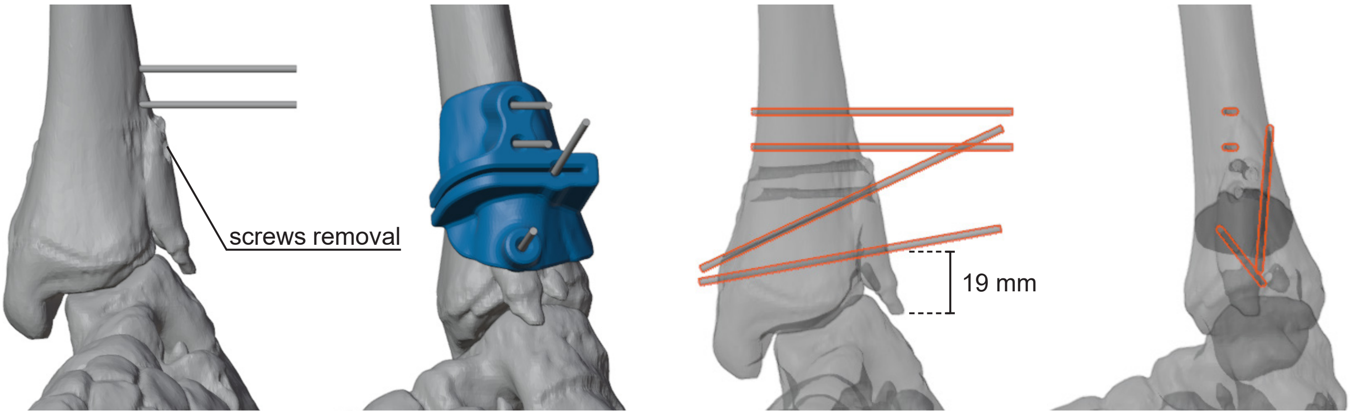

Osteotomy, 25° varus correction, bone wedge insertion

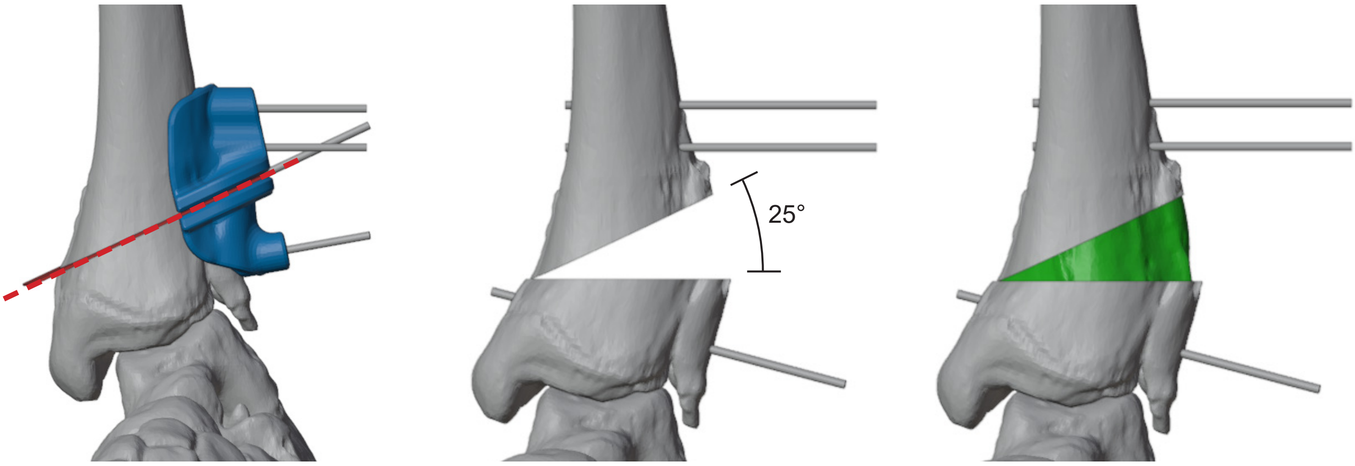

Fixation with 2 cannulated 3,5 mm screws

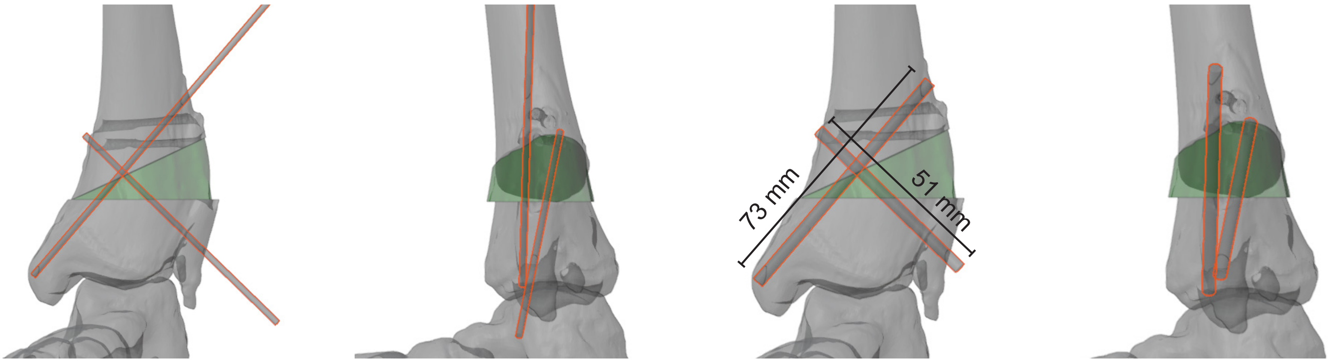

Supplement: Supplementary file 1 [file jpm-13-01664-s001.zip › jpm-2691682-supplementary.pdf]
